# ICMT deficiency ameliorates weight loss and mortality, but not tumor formation in a mouse model of liver cancer

**DOI:** 10.1186/s12964-026-02775-6

**Published:** 2026-03-11

**Authors:** Piotr Czarnota, Tomasz Gromowski, Anna Golda, Danuta Bryzek, Slawomir Lasota, Joanna Koziel, Mateusz Wilamowski, Jaroslaw Cisowski

**Affiliations:** 1 Department of General Biochemistry, Faculty of Biochemistry, Biophysics and Biotechnology, Jagiellonian University, Krakow, Poland; 2 Doctoral School of Exact and Natural Sciences, Jagiellonian University, Lojasiewicza 11, Krakow, 30- 348 Poland; 3 Department of Microbiology, Faculty of Biochemistry, Biophysics and Biotechnology, Jagiellonian University, Krakow, Poland; 4 Department of Cell Biology, Faculty of Biochemistry, Biophysics and Biotechnology, Jagiellonian University, Krakow, Poland

**Keywords:** Liver cancer, ICMT, Mouse models, Blood coagulation

## Abstract

Liver cancer often arises in the setting of chronic liver disease and remains a major global health challenge with limited effective treatments. Isoprenylcysteine Carboxyl Methyltransferase (ICMT) is an enzyme that catalyzes the methylation of prenylated proteins containing a CAAX motif, such as RAS, a modification thought to be essential for their function. Given its role in oncogenic signaling, ICMT has emerged as a potential therapeutic target in cancer. Notably, ICMT is overexpressed in human hepatocellular carcinoma (HCC), and previous studies have shown that *Icmt* deletion impairs *Braf*^V600E^-driven transformation in murine fibroblasts. In this study, we investigated the role of ICMT in liver cancer using a genetically engineered mouse model with hepatocyte-specific expression of *Braf*^V600E^, combined with deletion of *Trp53* and *Icmt*. We assessed the impact of *Icmt* loss on liver physiology, tumorigenesis, and global transcriptomic and proteomic landscapes. Expression of *Braf*^V600E^ led to progressive body weight loss, reduced survival, hepatomegaly, vascular congestion, liver fibrosis, and tumor development. These phenotypic changes were associated with widespread alterations in gene and protein expression, particularly affecting pathways related to cell differentiation, coagulation, and metabolism. Strikingly, liver-specific *Icmt* deletion ameliorated several pathological features, including weight loss, early mortality, fibrosis, and vascular abnormalities. It also partially normalized the transcriptomic and proteomic profiles. However*, Icmt* deletion did not prevent hepatomegaly or tumor formation. In conclusion, while ICMT deletion mitigates several deleterious effects of *Braf*^V600E^-driven liver pathology, it does not block tumorigenesis. These findings suggest that ICMT is not a suitable therapeutic target for liver cancer prevention or treatment, but may have potential as a target to alleviate liver disease-associated symptoms.

**Supplementary Information:**

The online version contains supplementary material available at 10.1186/s12964-026-02775-6.

## Introduction

Liver cancer remains one of the most common cancers worldwide and is associated with a high mortality rate. Although incidence has stabilized in men, it continues to rise by approximately 2% per year in women and younger adults [1], and predictions suggest 1.4 million new cases by 2040 [2]. Hepatocellular carcinoma (HCC) accounts for approximately 90% of all liver cancer cases [3]. Elevated HCC incidence is reported in regions with high prevalence of hepatitis B and C, as well as with high rates of obesity and type 2 diabetes mellitus [4, 5]. HCC usually develops on the background of chronic liver disease, such as fibrosis, cirrhosis [6], and non-alcoholic fatty liver disease [7]. Mortality rates in HCC remain high [2], highlighting the lack of efficient therapies, in particular for advanced HCC. Despite extensive efforts to develop new targeted drugs, the emergence of drug resistance poses a significant clinical challenge [8] underscoring the need for novel therapeutic targets.

Mitogen-Activated Protein Kinase/Extracellular Signal-Regulated Kinase (MAPK/ERK) pathway is frequently activated in liver cancer [9–11]. Elevated levels of phosphorylated ERK have been consistently observed in human HCC tissues compared to adjacent non-tumorous tissues [12] and were associated with increased cell proliferation, tumor aggressiveness, and therapeutic resistance [13, 14].

Isoprenylcysteine carboxyl methyltransferase (ICMT) catalyzes the methylation of isoprenylated C-terminal residues in proteins containing a CAAX motif—such as RAS, RHO, RAC, and RAB proteins—thereby promoting their proper localization to cellular membranes [15]. Given its role in post-translational modification of key signaling molecules, ICMT has emerged as a potential target for cancer therapy. Previous studies have shown that *Icmt* knockout in mice reduces disease burden in models of *Kras*^*G12D*^-driven myeloproliferative neoplasms and lung cancer [16] Notably, *Icmt* deletion in *Braf*^*V600E*^-expressing fibroblasts inhibited their transformed phenotype [17], suggesting that ICMT may influence BRAF^V600E^-driven oncogenesis even though BRAF^V600E^ is not a prenylated (CAAX) protein. These findings raise the possibility that ICMT could support oncogenic signaling indirectly or through other prenylated effectors.

ICMT expression is elevated in human HCC tissues, and its knockdown in HCC cell lines reduces proliferation and migration while inducing apoptosis, whereas ICMT overexpression has the opposite effect [18]. However, the in vivo consequences of Icmt deletion in a physiologically relevant context—particularly with endogenous gene expression and an intact immune system—remain poorly understood.

In this study, we utilized conditional gene modifications in the liver and demonstrated that *Icmt* deletion did not prevent BRAF^V600E^-induced hepatomegaly and HCC development, but significantly improved survival and reduced body weight loss. Transcriptomic and proteomic analyses revealed extensive changes in gene and protein expression, including alterations in pathways related to metabolism, differentiation, and coagulation. Mechanistically, Icmt deletion alleviated hepatic vascular congestion. Our findings suggest that while ICMT inhibition may not be effective as a direct anti-tumor strategy for HCC, it could have therapeutic value in reducing vascular pathology associated with liver disease.

## Materials and methods

### Mice

Mice were housed under specific pathogen-free conditions in ventilated cages in a temperature-controlled environment with 14-/10-h light/dark cycle. DNA was extracted from tail biopsies using the KAPA Mouse Genotyping Kit (KAPA Biosystems) according to the manufacturer’s instructions, and genotyping was carried out as previously described [17, 19, 20]. To generate compound mutant mice, albumin Cre (*Alb-Cre*) transgenic mice [19], were crossed with mice carrying a conditional *Braf* knock-in allele (*Braf*^*CA*^) [20], conditional *Trp53* knockout allele (B6.129P2-*Trp53*^*tm1Brn/J*^) [21], and conditional *Icmt* knock-out allele (*Icmt*^*flx*^) [17].

### Tissue processing, IHC staining and immunofluorescence

Organs were fixed in formalin, dehydrated and embedded in paraffin. Sections (4 µm thick) were deparaffinized and stained with hematoxylin & eosin (H&E) for histological evaluation or with picrosirius red for collagen detection. Images were acquired at 100 × magnification (Leica DMC5400, Leica Microsystems). Collagen content was quantified using ImageJ software. IHC for α 1 Fetoprotein (AFP) was done using a VECTASTAIN® Elite® ABC-HRP Kit and DAB Peroxidase Substrate (PK-6101 and SK-4100, respectively, Vector Laboratories) according to manufacturer’s instructions. Sections were blocked with 4% normal goat serum and then incubated with antibodies against AFP (ab46799, Abcam, 1:5000) overnight. For immunofluorescence, tissue sections were blocked with 5% normal goat serum, permeabilized with 0.1% Triton X-100 for 5 min, and incubated with primary antibodies against ApoA-1 (ab227455, Abcam, 1:500) or fibrinogen beta chain (ab27913, Abcam, 1:100) for 2 h at room temperature. Following washes, slides were incubated with goat anti-rabbit Alexa Fluor 488-conjugated secondary antibody (Cell Signaling Technology, 1:1000) for 1 h. Nuclei were stained using ProLong Antifade Mounting Medium with DAPI (Thermo Fisher Scientific). Images were acquired with a ZEISS Axio Observer 7 fluorescence microscope using ZEN software.

### Transcriptome sequencing

Total RNA was isolated and purified from ~ 10 mg of frozen murine livers using Trizol (Invitrogen, Carlsbad, CA, USA) according to the manufacturer’s procedure. RNA quality and quantity were assessed using NanoDrop 2000 (NanoDrop, Wilmington, DE, USA) and Agilent Bioanalyzer 2100 (Agilent Technologies, Santa Clara, CA, USA). Stranded mRNA libraries were prepared using Illumina’s Stranded mRNA Prep Kit and sequenced on an Illumina NovaSeq X Plus (Novogene, Cambridge, United Kingdom) with a paired-end 2 × 150 bp read length. Construction and sequencing of RNA-seq libraries were performed by the Novogene Company. The data received from Novogene Company was subjected to a process of trimming and preliminary analysis of the quality of readings.

Data analysis was performed using Galaxy web European server (https://usegalaxy.eu/). FastQC (http://www.bioinformatics.babraham.ac.uk/projects/fastqc, ver. 0.12.1) was used for quality control of concatenate reads. Alignment of paired-end reads to mouse (*Mus musculus*) build-in reference genome (mm10) was performed using HISAT2 program (ver. 2.2.1) [22]. Read counts were generated using FeatureCounts program (ver. 2.0.8) [23]. Differential gene expression analysis was performed with edgeR program (ver. 3.36) [24]. with multiple testing correction by the Benjamini–Hochberg method. Genes with adjusted p < 0.05 were considered significantly differentially expressed.

Selected genes were mapped to Biological Process (BP) Gene Ontology (GO) terms using ShinyGO 0.82 (https://bioinformatics.sdstate.edu/go/) [25]. Venn diagrams were generated with InteractiVenn tool (https://www.interactivenn.net/index2.html) [26]. Heatmaps were created with Galaxy, while volcano plots were generated in R using ggplot2 (v3.5.1). RNA-seq data are available under Gene Expression Omnibus (GEO) accession number GSE297390.

### Sample preparation and LC–MS/MS measurement

Protein lysates were sonicated for 7 min (30 s/30 s on/off cycle) using Bioruptor Pico (Diagenode), then centrifuged and supernatants were collected. Protein concentrations were measured with the Pierce Detergent-Compatible Bradford Assay (Thermo Fisher Scientific). For proteomic analysis, 20 µg of total protein was prepared using the SP3 protocol [27]. Peptides were suspended in 0.1% formic acid (FA) and 250 ng of peptide mixture was injected for the measurement with Orbitrap Astral mass spectrometer coupled to Vanquish Neo UHPLC (both Thermo Fisher Scientific). UHPLC was operated in a direct injection mode. Peptides were separated using Aurora Ultimate XT 25 cm × 75 µm C18 column (IonOpticks) in 60 min gradient of acetonitrile (ACN) at a flow rate of 0.5 µl/min at 55 °C. The gradient of ACN consisted of 3 major steps (buffer A: 0.1% FA, buffer B: 80% ACN, 0.1% FA): 2%−10% B 10 min, 10%−25% B 32 min, 25%−45% B 18 min. The eluting peptides were analyzed with Orbitrap Astral mass spectrometer using DIA method. Data were collected within 200 windows of 3-Th for the precursors from the mass range of 380–980 m/z. The maximum injection time (IT) was set to 5 ms and normalized AGC target to 500%. The isolated ions were fragmented with the normalized collision energy set to 25%. Astral scan range was 150–2000 m/z. Additionally, full-MS spectra were acquired every 0.6 s with the resolution of 240,000, scan range of 380–980 m/z, maximum IT of 3 ms and normalized AGC target of 500%.

### LC–MS/MS data analysis

Raw data were analyzed in DIA-NN 2.0.2 [28]using in silico DIA-NN predicted spectral library from mouse reference proteome (UniProtKB, 21 755 sequences, downloaded on Feb 2025). The DIA-NN spectral library building as well as search were performed using the following settings: protease – Trypsin/P, missed cleavages – up to 1, maximum number of variable modifications – 2, modifications – C carbamidomethylation, N-term M excision, M oxidation, protein N-terminal acetylation, peptide length range – 7–30, precursor charge range – 2–4, mass accuracy – 10 ppm, MS1 accuracy – 4 ppm, scan window – 7, scoring – peptidoforms, proteotypicity – genes, machine learning – NNs (cross-validated), quantification strategy – QuantUMS (high precision), cross-run normalization – RT-dependent, library generation – IDs, RT & IM profiling, speed and RAM usage – optimal results. MBR and protein inference were enabled. The resulting pg_matrix was filtered for protein groups that satisfy Global.PG.Q.Value threshold of 0.01. The list of protein groups that meet this criterion was obtained by filtering the main output file in Rstudio (v2024.12.1 + 563). Then, cRAP proteins were excluded. Data matrix was further processed in Perseus (v2.0.11.0) [29]. Protein intensity values were log2 transformed and matrix was filtered for proteins that had a minimum 80% of valid values in at least one group. Remaining missing values were imputed by constant low value. Differential proteins were identified using Student’s t-test with Benjamini–Hochberg FDR set to 0.01; threshold q-value < 0.01 and |Fold change|> 2. LCS/MS data have been deposited at the MassIVE database, accession number PXD064401.

### Coomassie blue staining

Twenty µg of denatured total liver protein extracts (in Laemmli buffer) were resolved in 12% SDS-PAGE. After electrophoresis, the gel was heated in water and stained with a 0.5% Coomassie brilliant blue G-250 solution solution (prepared in 40% methanol/10% acetic acid) for 15 min and heated again in water. After thorough washing the gel was imagined in a ChemiDoc detector (Bio-Rad). Band for identification by MS was cut-out using a sterile scalpel.

### Protein identification from gel band—LC–MS/MS analysis

LC–MS/MS identification was performed as described in [30] with minor modifications. Spectra were searched against the SwissProt *Mus musculus* database with the following parameters: enzyme – trypsin; missed cleavages – up to 1; fixed modifications – carbamidomethyl (C); variable modifications – oxidation (M), acetyl (protein N-term); precursor mass tolerance – 10 ppm; fragment mass tolerance – 20 mmu. Target Decoy PSM Validator was applied with the maximum false discovery rate (FDR) of 0.01.. LCS/MS data have been deposited at the MassIVE database, accession number PXD073274.

### Immunoblotting

Cells were washed with ice-cold PBS and total protein extracts were prepared using RIPA buffer containing proteinase and phosphatase inhibitors (Roche Diagnostics). Twenty µg of protein was resolved on SDS-PAGE and transferred to membranes. After blocking with 5% skim milk, membranes were incubated overnight at 4 °C with primary antibodies against: Apolipoprotein A1 (ApoA1, ab227455, Abcam, 1:2000), ICMT (17,511–1-AP, Proteintech, 1:1000), Fibrinogen beta chain (ab27913, Abcam, 1:1000), and α-Tubulin as a loading control (CP06, Meck Millipore, 1:2000) at 4 °C overnight. After washing, membranes were incubated with HRP-conjugated secondary antibodies (#7074 and #7076, Cell Signaling Technology, 1:2000) for one hour at RT. Signal was detected using ECL Select (GE Healthcare) and imaged on a ChemiDoc system.

### Statistical analysis

GraphPad Prism version 8.01 was used for analyses. Survival comparison was done using Log-rank (Mantel-Cox) test with correction for multiple comparisons. Statistical comparisons between groups were done using Student’s t-test. Multiple groups were compared using one-way ANOVA with Tukey’s post-hoc test. Data are presented as mean ± S.E.M. Comparisons were considered statistically significant when P < 0.05 (* *P* < 0.05; ** *P* < 0.01; *** *P* < 0.001; **** *P* < 0.0001).

## Results

### *Icmt* deletion in mouse liver rescues body weight loss and mortality caused by expression of *Braf*^*V600E*^

To investigate the impact of ICMT on liver tumorigenesis, we crossed mice with a knock-in expression of a liver-specific *Albumin-Cre*, with mice containing *Braf*^*CA/*+^ allele, conditional knockout of *Trp53* gene (*Trp53*^*flx*^), and conditional knockout of *Icmt* gene (*Icmt*^*flx*^*)*. This breeding strategy yielded four genotypes: *Alb-Cre/Braf*^*V600E*^*/Trp53*^*flx/flx*^*/Icmt*^+/+^ (designated as ABpI^+/+^)*, Alb-Cre/Braf*^*V600E*^*/Trp53*^*flx/flx*^*/Icmt*^*flx/+*^ (designated as ABpI^F/+^), *Alb-Cre/Braf*^*V600E*^*/Trp53*^*flx/flx*^*/Icmt*^*flx/flx*^ (designated as ABpI^F/F^) and control siblings lacking the expression of *Braf*^*CA*^ allele, Alb-Cre/Trp53^*flx/flx/*^*/Icmt*^*flx/+*^ (designated as Ctrl). ABpI^+/+^ and ABpI^F/+^ mice were born at expected Mendelian ratios, however, ABpI^F/F^ mice were born at approximate frequency of 1 in 100, and only three mice with this genotype were obtained, all females.

**Fig. 1:**
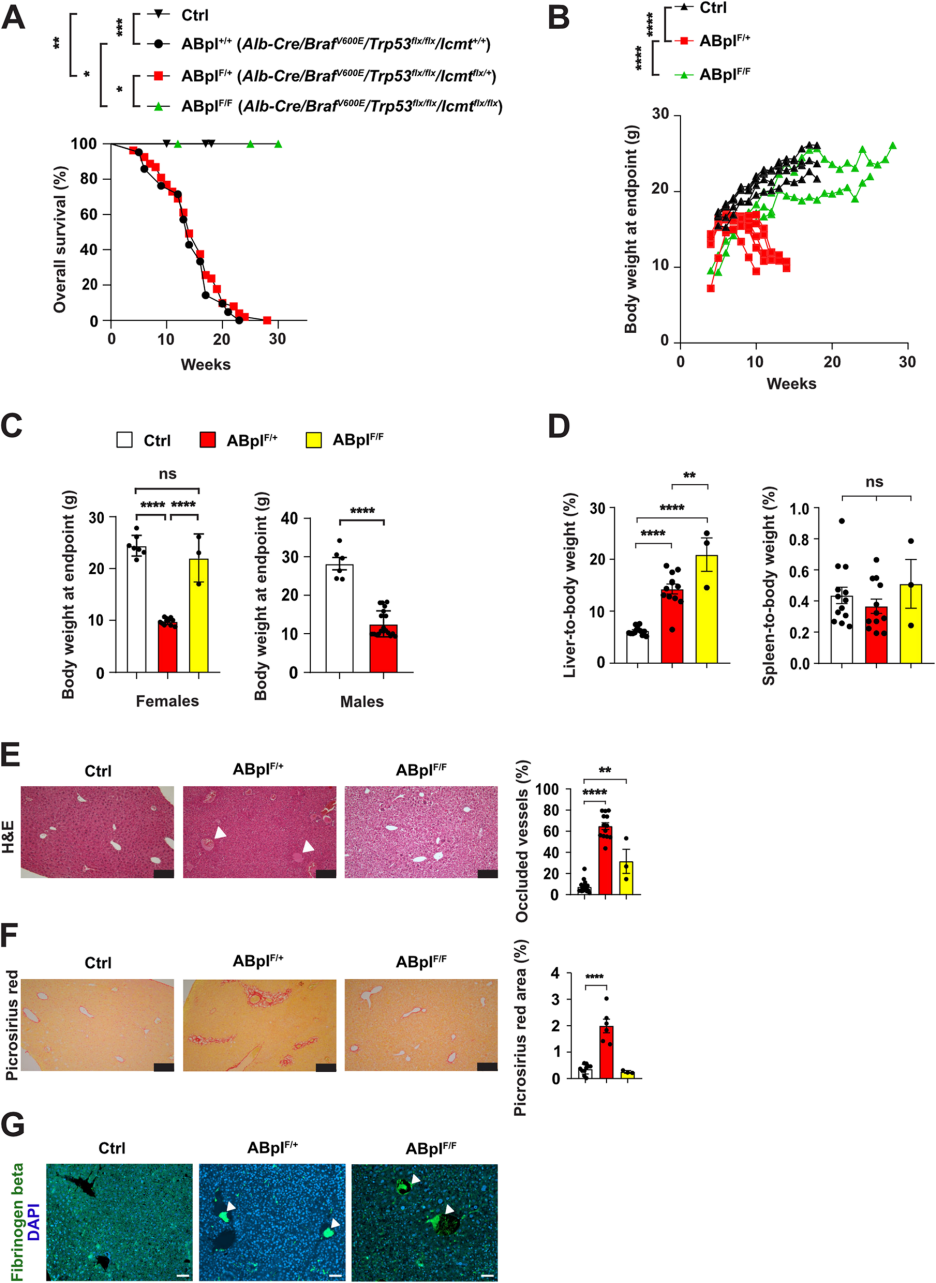
Liver-specific deletion of the *Icmt* gene rescues decreased viability, body weight loss and histopathologic abnormalities induced by *Braf*^*V600E*^ and deletion of *Trp53* gene. **A** Overall survival of Ctrl (n = 10), ABpI^+/+^ (*n* = 21), ABpI^F/+^ (*n* = 51), and ABpI^F/F^ (*n* = 3) mice. **B** Body weight changes over time in individual mice grouped by genotype; Ctrl (*n* = 5), ABpI^F/+^ (*n* = 7), and ABpI^F/F^ (*n* = 3) mice. **C** Endpoint body weight of female (left): Ctrl (*n* = 7), ABpI^F/+^ (*n* = 12), and ABpI^F/F^ (*n* = 3), and male mice (right): Ctrl (*n* = 6), ABpI^F/+^ (*n* = 19). **D** Liver (left) and spleen (right) weights as a percent of total body weight at endpoint: Ctrl (*n* = 13), ABpI^F/+^ (*n* = 12), and ABpI^F/F^ (*n* = 3) mice. **E** Representative H&E-stained liver sections from Ctrl (*n* = 18), ABpI^F/+^ (*n* = 13), and ABpI^F/F^ (*n* = 3) mice. White arrowheads indicate congested vessels. Right, quantifications of congested vessels. Scale bars, 100 µm. **F** Representative images of picrosirius red-stained liver sections from *Ctrl* (n = 10), ABpI^F/+^ (*n* = 6), and ABpI^F/F^ (*n* = 3) mice. Right, quantifications of stained area. Scale bars, 100 µm. **G** Representative images showing Fibrinogen beta chain (green) localization in liver sections. Nuclei were counterstained with DAPI (blue). White arrowheads indicate stained areas within blood vessels. Scale bars, 50 µm

We monitored body weight weekly from weaning until spontaneous death or euthanasia due to weight loss or poor condition. All ABpI^+/+^ and ABpI^F/+^ mice had died or were euthanized by weeks 23 and 28, respectively; with a median survival time of both ABpI^+/+^ and ABpI^F/+^ mice approximating 14 weeks (Fig. 1 A), which suggests that the ICMT expression derived from a single allele may be sufficient to sustain its function, especially in hepatocytes, the majority of which (~ 90%) are polyploid in mice [31]. Moreover, there was no statistically significant difference in survival times between males and females in ABpI^+/+^ group (median survival time 16,5 vs 14 weeks, respectively) or in the ABpI^F/+^ group (median survival time 13 vs 16 weeks, respectively) (Supplementary Fig. 1A.). Since ABpI^+/+^ and ABpI^F/+^ mice displayed similar phenotypes and survival, we focused subsequent analyses on Ctrl, ABpI^F/+^ and ABpI^F/F^ mice.

Initially, all groups gained weight regardless of sex. However, from around week 7 post-partum, ABpI^F/+^, but not Ctrl or ABpI^F/F^ mice, began losing weight (Fig. 1B) and developed signs of illness (hunched posture, limping), whereas Ctrl and ABpI^F/F^ mice continued to gain weight at similar rates (Fig. 1B) and did not develop signs of distress or visible signs of disease. At the endpoint, the body weights of ABpI^F/+^ females and males were roughly half the body weight of their respective controls, while the body weights of ABpI^F/F^ females were roughly twice as high as those of ABpI^F/+^ females, and were comparable Ctrl females (Fig. 1C). At necropsy, we found that livers of ABpI^F/+^ and ABpI^F/F^ mice had enlarged livers compared to Ctrl mice (Fig. 1D, left, and Supplementary Fig. 1B); spleen sizes, however, were not statistically different between genotypes (Fig. 1D, right), suggesting that differences in liver or spleen mass alone cannot account for the increased body weight and survival observed in ABpI^F/F^ mice. As expected, ICMT protein levels were markedly reduced in the livers of ABpI^F/F^ compared to the livers of ABpI^F/+^ and Ctrl mice (Supplementary Fig. 1 C), and PCR genotyping confirmed liver-specific activation of *Braf*^*V600E*^ and deletion of *Trp53* (Supplementary Fig. 1 D).

Histological analysis of H&E-stained liver sections showed extensive vascular congestion with eosinophilic deposits in ABpI^F/+^ mice, particularly in periportal areas (Fig. 1E). Moreover, picrosirius red staining revealed increased periportal collagen deposition indicative of fibrosis (Fig. 1F). Notably, both vascular congestion and fibrosis were markedly reduced in ABpI^F/F^ mice (Fig. 1E and F). We hypothesized that the vascular congestion resulted from blood clots obstructing vessels. To verify this hypothesis, we stained for fibrinogen beta chain (FGB), a key component of clots formed following thrombin cleavage. FGB was absent in vessel lumens of Ctrl mice but clearly present in blood vessels of ABpI^F/+^ and ABpI^F/F^ mice (Fig. 1G). In addition, less FGB could be detected in the parenchyma of ABpI^F/+^ and ABpI^F/F^ mice, as compared to Ctrl mice (Fig. 1G). Moreover, immunoblot analysis revealed overall higher expression of FGB in ABpI^F/+^ mice as compared to Ctrl and ABpI^F/F^ mice (Supplementary Fig. 1E). As expected, liver extracts from ABpI^F/+^ mice contained and additional band slightly below a full length FGB protein, which is consistent with FGB being cleaved by thrombin protease (which chops out 14 aa peptide) and contributing to fibrin generation and clot formation [32].

Together, these results demonstrate that ABpI^F/+^ mice exhibited reduced body weight, shortened lifespan, hepatomegaly, liver fibrosis, and vascular occlusion—likely due to disrupted hemostasis. Moreover, *Icmt* deletion ameliorated all these phenotypes except hepatomegaly.

### *Icmt* deletion does not prevent liver tumor formation

We found that approximately 30% of ABpI^F/+^ mice had at least one visible surface liver tumor (1–2 mm in diameter; Supplementary Fig. 2 A). The relatively low tumor frequency likely reflects early mortality before overt tumor development. In contrast, despite improved survival, all three ABpI^F/F^ mice developed liver tumors, with burden correlating with age (Supplementary Fig. 2B). A 12-week-old ABpI^F/F^ mouse had multiple small tumors, while 20- and 30-week-old mice developed large (~ 1 cm) tumors (Supplementary Fig. 2B). Upon microscopic examination, the tumors exhibited a pushing growth pattern against the surrounding tissue (Supplementary Fig. 2 C) and were positive for α 1 Fetoprotein (AFP), a marker of HCC (Supplementary Fig. 2D). Additionally, 19% of ABpI^F/+^ mice developed subcutaneous metastatic tumors with sarcomatoid features and collagen deposition (Supplementary Fig. 2E).

Taken together, these results demonstrate that *Icmt* deletion does not prevent liver tumor formation.

## Transcriptomic and proteomic analyses reveal major gene expression changes induced by *Braf*^*V600E*^ expression and deletion of *Icmt*

**Fig. 2:**
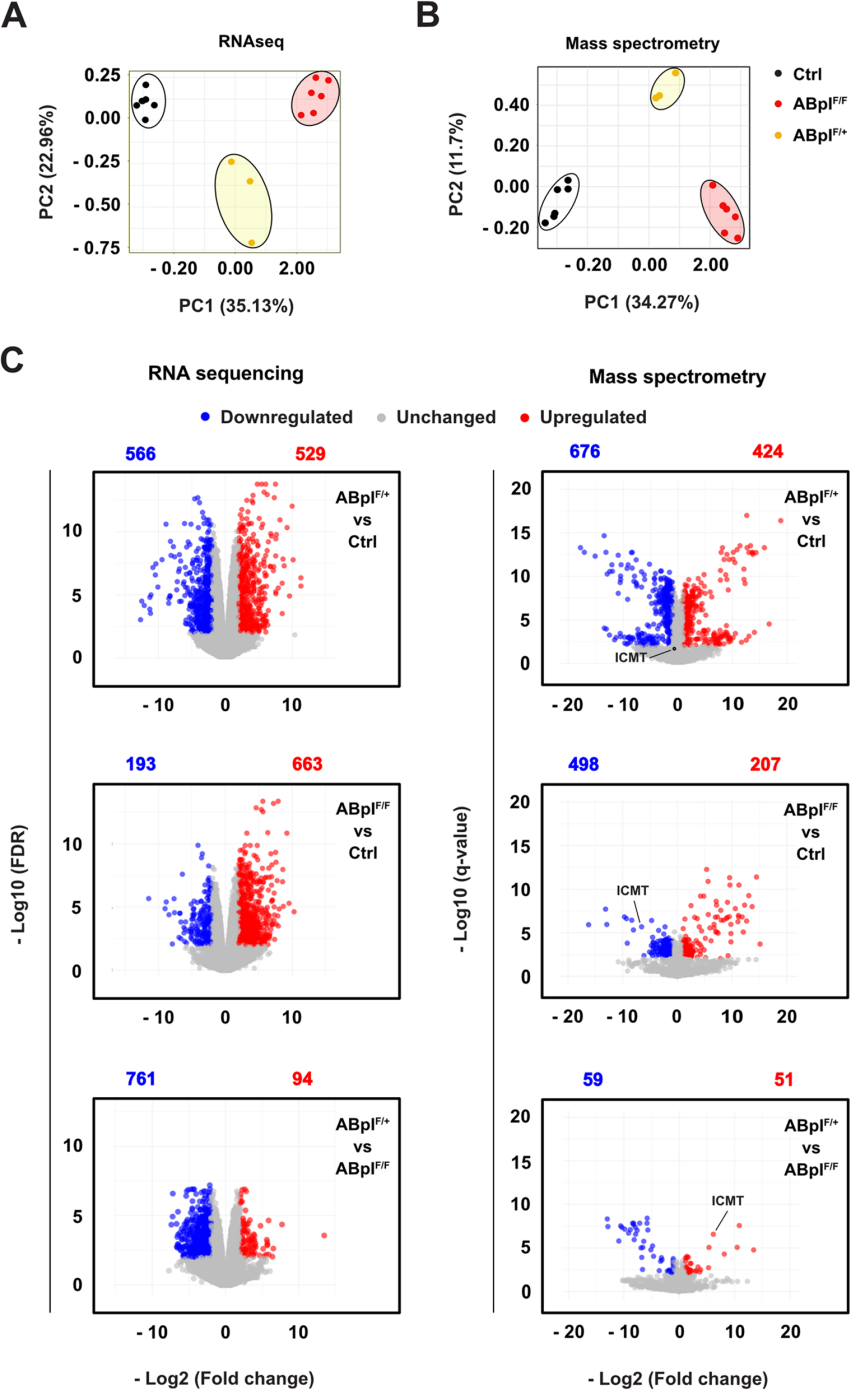
Transcriptomic and proteomic data identify three groups based on mouse genotypes. Principal component analysis of differentially-expressed genes obtained from **A** RNAseq (left) and **B** mass spectrometry (right) analyses. **C** Volcano plots showing significantly differentially expressed genes (left) and proteins (right) between indicated genotypes; downregulated (blue), unchanged (grey), and upregulated (red). Numbers above plots indicate total differentially expressed genes or proteins. The position of the ICMT protein is indicated on mass spectrometry plots

To explore the molecular mechanisms underlying observed phenotypes, we performed RNA sequencing (RNAseq) and mass spectrometry (MS) on liver tissue (free of visible tumors) from six Ctrl; six ABpI^F/+^ and three ABpI^F/F^ mice (all females to reduce variability) to determine differentially expressed genes (DEGs) and proteins (DEPs), respectively. Differential expression analysis used thresholds of fold change (FC) ≥ 2.0 or ≤ −2.0 and adjusted *p* < 0.01 (Supplementary Tables 1 and 2, respectively). Principal Component Analysis (PCA) showed clear separation of mice into three groups, depending on their genotypes (Fig. 2A and B). The separation between Ctrl and ABpI^F/+^ groups was largest, with the position of ABpI^F/F^ group shifted slightly towards the Ctrl group suggesting that there were fewer DEGs and DEPs in ABpI^F/F^ group than in ABpI^F/+^ group, as compared to the Ctrl group (Fig. 2A and B). Heatmaps of z-score–normalized gene and protein expression further confirmed distinct clustering by genotype (Supplementary Fig. 3 A and B).

We identified 1,095 DEGs (529 upregulated, 566 downregulated) in ABpI^F/+^ vs Ctrl, 856 DEGs in ABpI^F/F^ vs Ctrl (663 upregulated, 193 downregulated), and 855 DEGs in ABpI^F/+^ vs ABpI^F/F^ (94 upregulated, 761 downregulated) (Fig. 2C; Supplementary Fig. 3 C). MS identified 1,100 DEPs in ABpI^F/+^ vs Ctrl (424 upregulated, 676 downregulated), 705 DEPs in ABpI^F/F^ vs Ctrl (207 upregulated, 498 downregulated), and 110 DEPs in ABpI^F/+^ vs ABpI^F/F^ (51 upregulated, 59 downregulated) (Fig. 2C; Supplementary Fig. 3D). These data show that *Braf*^*V600E*^ expression induces widespread transcriptional and proteomic changes.

### Differentiation and metabolism are the most affected biological processes

**Fig. 3:**
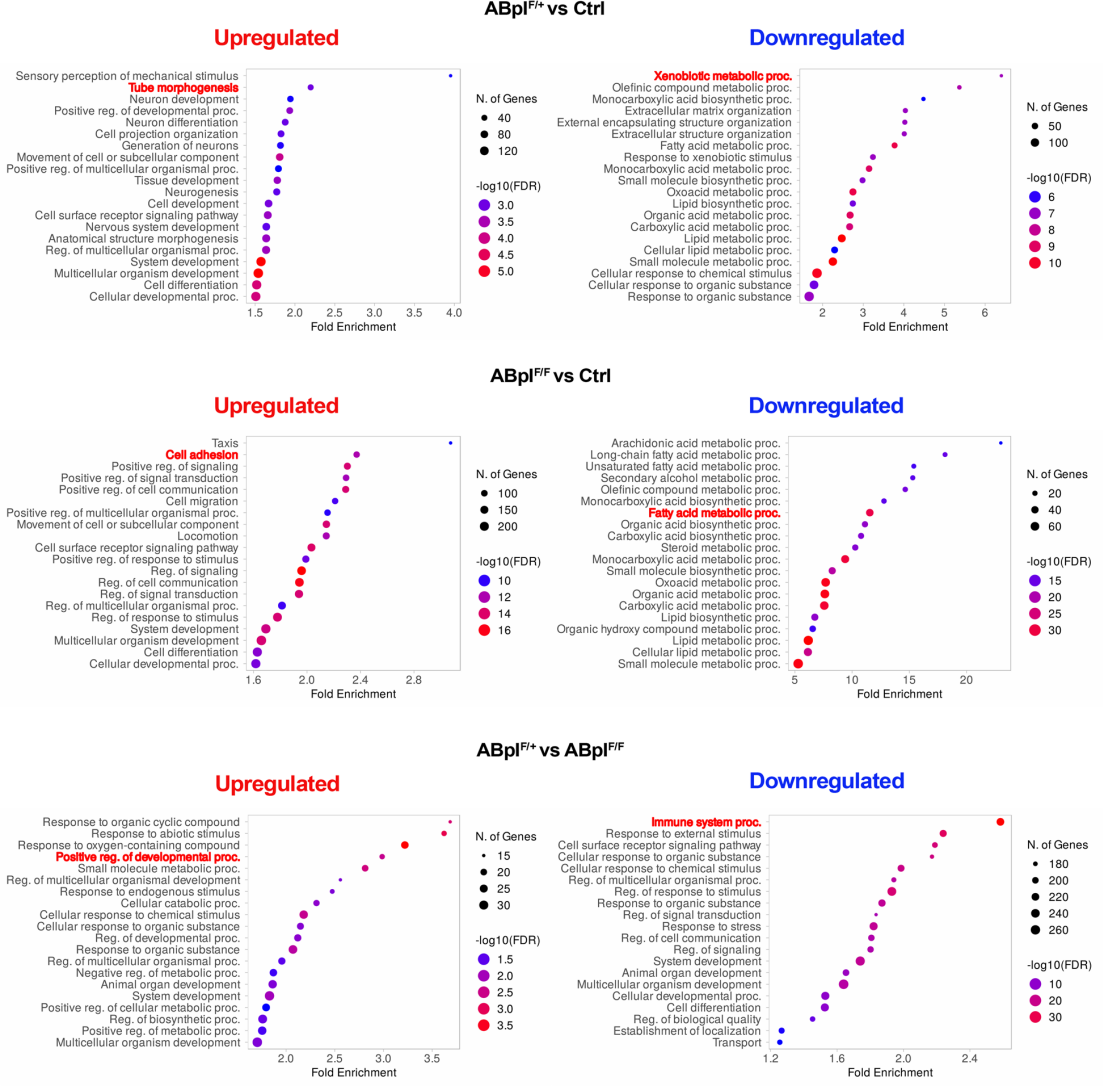
Significantly enriched biological processes identified by transcriptomic analysis. Gene ontology terms of upregulated (left) and downregulated (right) biological processes from RNA sequencing comparisons. Dot size represents the number of genes; dot color indicates FDR. Processes also shown as heatmaps in Supplementary Fig. 4 are highlighted in bold red

Gene Ontology (GO) analysis of DEGs (Fig. 3) revealed that upregulated biological processes in the livers of ABpI^F/+^ mice as compared to the Ctrl livers were mostly related to differentiation (e.g. tube morphogenesis, positive regulation of developmental processes, tissue and cell development, cell differentiation), whereas downregulated biological processes were mostly related to metabolism (e.g. xenobiotic metabolic processes, fatty acid metabolic processes, lipid metabolic processes, small molecule biosynthetic processes). Processes most significantly upregulated in the livers of ABpI^F/F^ mice as compared to the livers of Ctrl mice, in addition to differentiation, were related to cell movement and intracellular signaling (e.g. taxis, cell adhesion, cell migration, regulation of cell signaling and communication), whereas downregulated biological processes, akin to ABpI^F/+^ mice, were mostly related to metabolism (e.g. arachidonic acid metabolic processes, fatty acid metabolic processes, organic acid metabolic processes, steroid metabolic processes, lipid biosynthetic processes, small molecule metabolic processes). Taken together, these data suggest that the major role of *Braf*^*V600E*^ oncogene expression in the livers of both ABpI^F/+^ and ABpI^F/F^ mice is deregulation of differentiation and resultant downregulation of metabolic processes.

When comparing livers from ABpI^F/+^ mice to livers from ABpI^F/F^ mice, we found that biological processes most significantly upregulated were related to metabolism and differentiation (e.g. response to organic cyclic compounds, response to abiotic stimulus, response to oxygen-containing compounds, positive regulation of developmental processes, small molecule metabolic processes), while immune- and development-related processes were downregulated (e.g. innate immune response, response to external stimulus, cell surface receptor signaling pathway, system development, cell differentiation). Genes contributing to the representative enriched pathways are summarized in Supplementary Table 3, and heatmaps of DEGs for representative biological processes are presented in Supplementary Fig. 4.

**Fig. 4:**
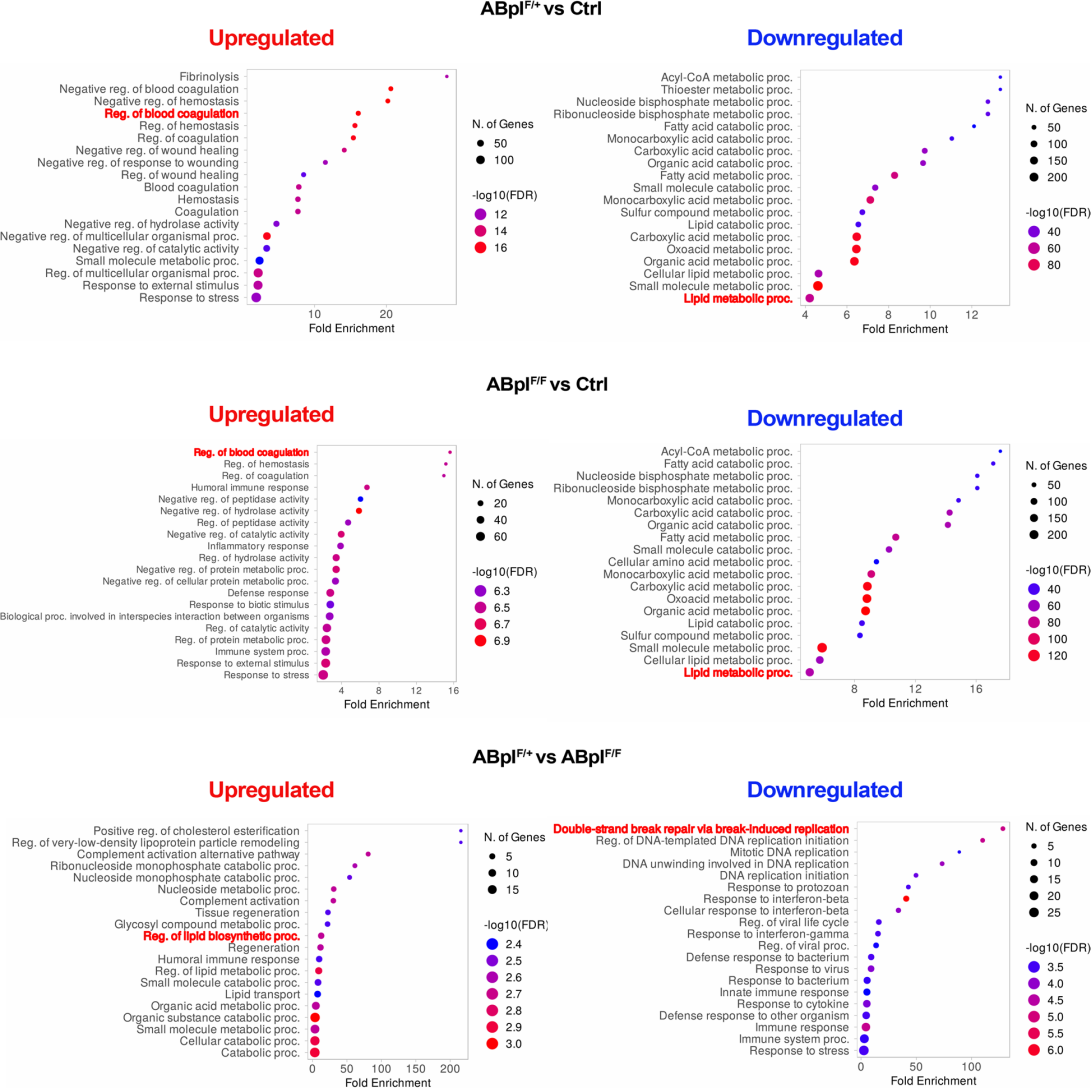
Significantly enriched biological processes identified through proteomic analysis. Gene ontology terms of upregulated (left) and downregulated (right) biological processes from mass spectrometry data. Dot size represents the number of proteins; dot color indicates FDR. Processes also shown as heatmaps in Supplementary Fig. 5 are highlighted in bold red

GO analysis of DEPs (Fig. 4) revealed that blood coagulation and hemostasis were the most significantly upregulated biological processes in ABpI^F/+^ mice as compared to the livers of Ctrl mice, and ABpI^F/F^ mice as compared to the livers of Ctrl mice (e.g. regulation of blood coagulation, regulation of hemostasis). In ABpI^F/F^ mice upregulated processes included immune and inflammatory responses (e.g. inflammatory response, defense response, immune system processes). Downregulated processes were consistently related to metabolic pathways (e.g. acyl-CoA metabolic processes, fatty acid metabolic processes, small molecule catabolic processes, lipid metabolic processes). Comparing the livers of ABpI^F/+^ and ABpI^F/F^ mice revealed upregulated metabolic and immune function-related processes (e.g. positive regulation of cholesterol esterification, complement activation, nucleoside metabolic processes), while downregulated biological processes related to the DNA repair pathway, mitosis and immune response (e.g. double strand break repair, mitotic DNA replication, response to interferon beta, response to cytokine). Proteins contributing to the representative enriched pathways are summarized in Supplementary Table 4, and heatmaps of DEPs for representative biological processes are presented in Supplementary Fig. 5.

In summary, *Braf*^*V600E*^ expression profoundly disrupted differentiation, metabolism, and hemostasis in the liver—effects partially reversed by *Icmt* deletion. Moreover, transcriptomic data highlighted altered cell differentiation, while proteomic analysis pointed to abnormal coagulation, consistent with observed vascular occlusions. Metabolic pathways were significantly downregulated in both datasets.

### ApoA1 overexpression in ABpI^F/+^ mice may contribute to vascular congestion

**Fig. 5:**
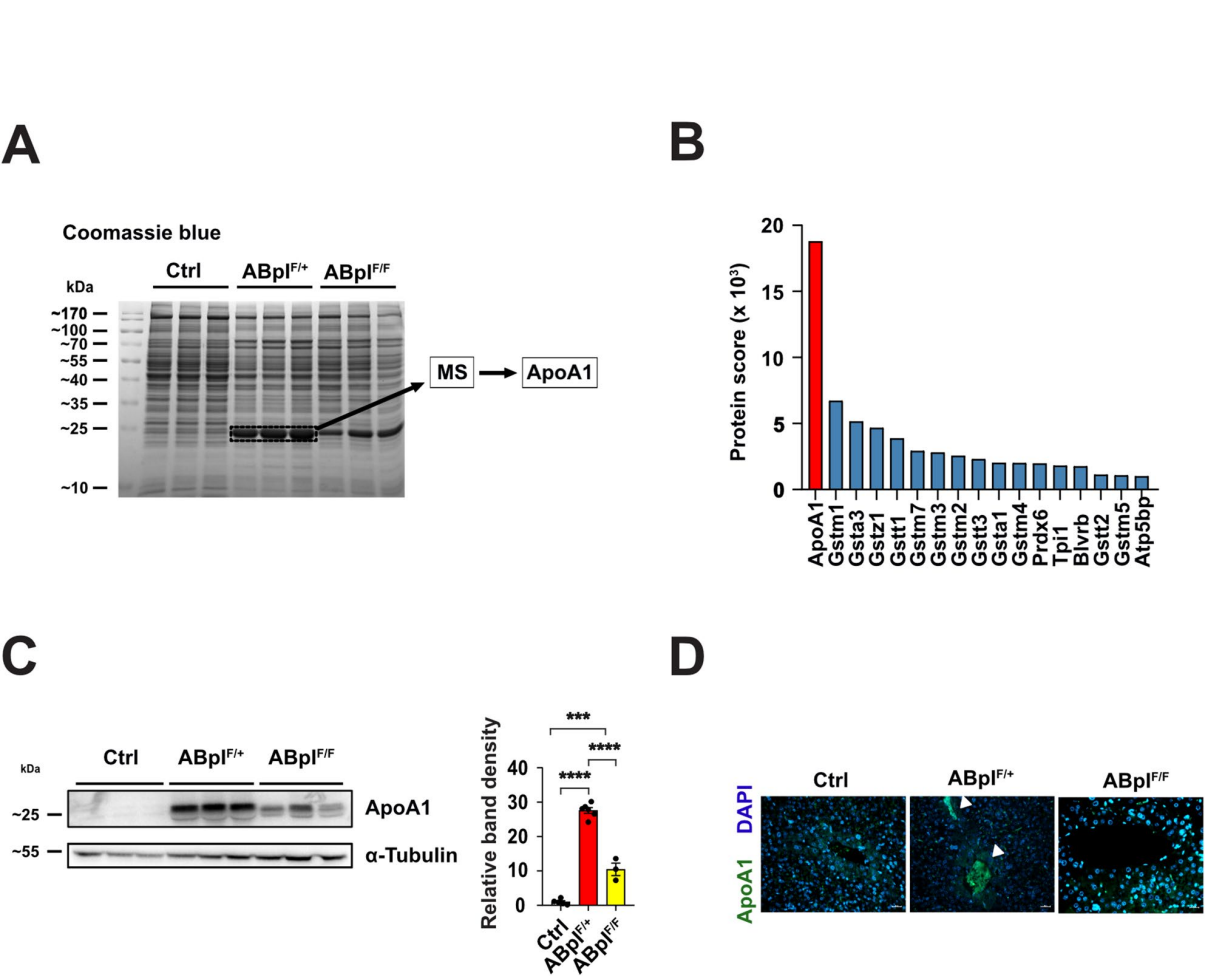
ApoA1 protein is overexpressed in ABpI^F/+^ mice and localizes to congested vessels. **A** Twenty µg of total liver protein from each genotype (*n* = 3) was run on a 12% acrylamide gel and stained with Coomassie blue. The boxed protein band was excised for mass spectrometry identification. **B** Bar graph of proteins with enrichment scores ≥ 1000 from the analyzed band. **C** Immunoblot detecting ApoA1 protein in liver extracts of mice with respective genotypes. Right, quantification of bands density relative to α-Tubulin. **D** Immunofluorescent staining of liver sections; scale bar, 50 µm. White arrowheads indicate ApoA1 staining within congested blood vessels

Coomassie staining of liver protein extracts revealed a prominent 25 kDa band in ABpI^F/+^ mice, less intense in ABpI^F/F^, and weak in Ctrl mice (Fig. 5A). Mass spectrometry identified Apolipoprotein A1 (ApoA1) as the major component of this band (Fig. 5B, and Supplementary Table 5). Immunoblotting confirmed ~ 30-fold higher ApoA1 expression in ABpI^F/+^ mice compared to Ctrl, and ~ threefold lower in ABpI^F/F^ mice compared to ABpI^F/+^ mice (Fig. 5C). Congruently, ApoA1 also contributed to the most significantly changed biological processes (positive regulation of cholesterol esterification and regulation of very-low-density lipoprotein particle remodeling) when comparing liver proteomes between ABpI^F/F^ vs Ctrl mice, and ABpI^F/+^ vs ABpI^F/F^ mice (Supplementary Table 4, and Supplementary Fig. 5). Other proteins in the same band included glutathione S-transferase isoforms of similar molecular weight (Fig. 5B and Supplementary Table 5). Given that ApoA1 is a major HDL component [33] and is secreted into circulation, we hypothesize it may accumulate in liver vasculature, as observed in ABpI^F/+^ and ABpI^F/F^ mice, and previously suggested [34]. Immunohistochemistry confirmed ApoA1 presence in both liver parenchyma and vessel-occluding material in ABpI^F/+^ and ABpI^F/F^ mice (Fig. 5D).

Thus, ApoA1 was significantly overexpressed in ABpI^F/+^ livers and accumulated in blood vessels, possibly contributing to congestion. *Icmt* deficiency reduced ApoA1 accumulation which may help explain the reduced vascular pathology and extended survival observed in ABpI^F/F^ mice.

## Discussion

This study aimed to elucidate the role of ICMT in liver cancer using a genetically engineered mouse model combining liver-specific expression of oncogenic *Braf*^*V600E*^, deletion of *Trp53*, and either one or two deleted alleles of the *Icmt* gene.

We found that ABpI^F/+^mice were initially healthy, however, starting from ~ 7 weeks of age, began to deteriorate rapidly, showing weight loss, poor condition, and reduced survival. Some mice suffered fatal intraperitoneal hemorrhages [35]. Consistent with prior reports [35–37], these mice also developed hepatomegaly. Histological examination showed widespread vascular congestion, likely due to thrombi, as the obstructing material contained cleaved fibrinogen and ApoA1. This aligns with studies linking BRAF mutations to platelet activation and thrombosis, which could be mitigated by anti-thrombotic treatments like aspirin [36]. We propose that vascular congestion impaired liver perfusion, increased local blood pressure, and promoted vessel rupture, contributing to weight loss and early mortality.

Remarkably, homozygous deletion of *Icmt* reversed many of these pathologies. ABpI^F/F^ mice showed normal weight gain and improved general health, with reduced vascular congestion and normalized fibrosis despite persistent hepatomegaly. These findings are consistent with our hypothesis that blood vessel congestion is the main contributor to health deterioration and mortality observed in ABpI^F/+^ mice. Moreover, fibrosis in ABpI^F/F^ mice was normalized to control values suggesting less tissue damage.

The mechanisms through which deletion of *Icmt* gene contributed to the improved disease phenotypes are unclear. BRAF^V600E^ is not a CAAX motif-containing protein and therefore is unlikely to be a direct substrate of ICMT. However, deletion of *Icmt* gene has previously been shown to block oncogenic transformation of murine fibroblasts induced by *Braf*^*V600E*^ expression[17], yet the mechanism remains unclear and requires further investigation.

The ICMT enzyme has been previously suggested as a viable target for liver cancer therapy; knockdown of the *ICMT* in human HCC cell lines using siRNA inhibited their growth, survival and migration, whereas ectopic expression of ICMT had an opposite effect [18]. Moreover, deletion of *Icmt* effectively ameliorated diseases phenotypes (reduced splenomegaly and growth factor-independent colony growth of splenocytes) in a mouse model of myeloproliferative diseases and lung cancer driven by expression of *Kras*^*G12D*^ [16]. In addition, deletion of *Icmt* in mouse embryonic fibroblasts blocked their transformation by *Braf*^*V600E*^ oncogene [17]. We found that, in the absence of *Icmt*, liver tumors developed with 100% penetrance; however, this observation should be interpreted with caution, as the number of mice analyzed was very small (three female mice), limiting the statistical power and generalizability of our findings. For this reason, it is difficult to assess whether *Icmt* deletion affected liver tumor development in any meaningful way. Moreover, the absence of viable male mice with the ABpI^F/F^ genotype precludes any conclusions regarding the impact of *Icmt* allele deletion on liver tumor development in males. It is possible that fibroblasts and hepatocytes differ in their requirements for ICMT in *Braf*^*V600E*^-induced oncogenic transformation. BRAF protein, in contrast to proteins such as KRAS, does not contain a CAAX motif and is therefore unlikely to be a direct target of ICMT. Moreover, and perhaps more importantly, hepatocytes are highly polyploid cells; up to 90% of rodent hepatocytes have been shown to be polyploid [31], which makes efficient deletion of the *Icmt* gene more challenging than in diploid cells, due to the higher number of alleles that must be excised by Cre recombinase to achieve complete gene inactivation. Consequently, we cannot exclude the possibility of incomplete *Icmt* deletion, with residual ICMT protein levels being sufficient to sustain tumor development.

Nevertheless, our findings question the validity of ICMT as a therapeutic target in HCC. Accordingly, an earlier report showed that *Icmt* deletion caused a dramatically accelerated tumor progression in a mouse model of *Kras*^*G12D*^-driven pancreatic adenocarcinoma [38].

Interestingly, sarcomatoid subcutaneous metastases with collagen deposition were observed only in ABpI^F/+^. Although we do not know at present which cell type they were derived from, it is tempting to speculate that cells residing in collagen-enriched areas around portal tracts might be implicated. For example, cholangiocarcinoma (malignancy derived from transformed cholangiocytes) was reported to generate sarcomatoid tumors in patients [39, 40]. The fact that we did not detect metastases in ABpI^F/F^ mice may be simply due to the low number of mice, the significantly lower periportal fibrosis in ABpI^F/F^ mice, or an anti-metastatic effect of *Icmt* deletion.

The lower-than-expected birth rate of ABpI^F/F^ mice may stem from the hypomorphic nature of the *Icmt*^*flx*^ allele (it is expressed at a lower level even in the absence of Cre-mediated excision) [41], and the known embryonic lethality of full *Icmt* knockout by E12.5 [42]. Therefore, partial downregulation of *Icmt* expression may cause substantial embryonic lethality as well, and raises the possibility that reduced *Icmt* expression after birth may contribute to the disease amelioration observed in ABpI^F/F^ mice.

In order to get insight into the molecular mechanisms of the observed phenotypes, we determined changes in transcriptomes and proteomes. PC analysis of both RNAseq and MS datasets revealed well separated clustering of mice depending on genotypes. The largest number of DEGs and DEPs were detected between ABpI^F/+^ vs Ctrl mice, followed by ABpI^F/F^ vs Ctrl mice, and ABpI^F/+^ and ABpI^F/F^ mice.

GO analysis of DEGs between ABpI^F/+^ and ABpI^F/F^ mice revealed that the upregulated processes were related to differentiation, adhesion, and signaling; whereas downregulated processes were related to metabolism. Comparison between ABpI^F/+^ and ABpI^F/F^ mice revealed that the upregulated processes were related to metabolism and downregulated process to immune system regulation. It is noteworthy, that *Sox9*, a marker of cells with progenitor characteristics forming ductular reaction [43, 44], was common among genes enriched in many of the differentiation-related processes (Supplementary Fig. 4, and Supplementary Table 3). On the contrary, several of the downregulated genes belonged to the cytochrome P450 family (*Cyp2* and *Cyp4*) (Supplementary Fig. 4 and Supplementary Table 3) contributing to the decreased metabolic abilities. This suggests that *Braf*^*V600E*^ expression might have caused hepatocyte dedifferentiation and decreased their metabolic properties, as previously demonstrated [45]. Conversly, *Icmt* deficiency could partially reverse this process.

GO analysis of DEPs between ABpI^F/+^ and Ctrl, and ABpI^F/F^ and Ctrl mice revealed that the upregulated processes were related to blood coagulation and hemostasis (both positive and negative), providing additional support for our hypothesis that abnormal coagulation underlies blood vessel congestion. In fact, fibrinogen chains alpha, beta, gamma, and coagulation factors: II (prothrombin), VII, and XI, were upregulated in ABpI^F/+^ mice as compared to Ctrl mice. However, these proteins were not upregulated in ABpI^F/F^ as compared to Ctrl mice (except for factor II), suggesting partial normalization of coagulation, consistent with lower percentage of congested vessels in ABpI^F/F^ mice. Our data support the idea, that chronic liver disease is more pro- than anti-thrombotic [46]. Moreover, this procoagulant state may contribute to liver fibrosis either through the formation of microthrombi leading to hypoxia and vascular injury, or through direct activation of stellate cells [46]. Moreover, we suggest that the lack of overlap in upregulated DEGs and DEPs between ABpI^F/+^ and Ctr mice, and ABpI^F/F^ and Ctrl mice may be caused by extensive accumulation of coagulation proteins independent of transcription, which overshadowed changes in proteins participating in differentiation-related processes. In agreement with RNAseq, the downregulated DEPs between ABpI^F/+^ and Ctrl, and ABpI^F/F^ and Ctrl mice were related to metabolism, with cytochrome P450 family members, CYP2 and CYP4 being among the most significantly downregulated.

Comparison between ABpI^F/+^ and ABpI^F/F^ mice revealed that the upregulated processes were mostly related to metabolism, confirming RNAseq data, and suggesting that *Icmt* deficiency may partially reverse metabolic impairment induced by *Braf*^*V600E*^ expression. The downregulated processes were related to DNA replication, DNA damage, mitosis and immune responses, possibly relating to the incipient tumorigenesis in ABpI^F/F^ mice.

Interestingly, accumulation of ApoA1—a key HDL component with known anti-inflammatory and tumor-suppressive functions [47]—was observed within congested vessels in ABpI^F/+^ mice. ApoA1 was shown to decrease tumor cell proliferation, increase apoptosis and reduce MAPK signaling [48]. In human HCC tissues *ApoA1* mRNA and protein levels were reduced [47, 49, 50], as were levels in sera from [51, 52]. ApoA1 is also used as a biomarker of HCC progression[48, 51]. Thus, ApoA1 protein was shown to play a tumor-suppressive role in HCC. The possibility that ApoA1 plays a tumor suppressive function in ABpI^F/+^ mice requires further investigation. While ApoA1 may play a protective role in tumorigenesis, its presence within clots suggests it could also contribute to vascular obstruction. Indeed, ApoA1 was previously found to associate with fibrin clots [34]. However, as ApoA1 is not known to functionally participate in blood clot formation, it likely plays a passive role in vascular congestion as a component of HDL particles, the concentration of which is significantly increased in the plasma of *Alb-Cre/Braf*^*V600E*^*/p53*^*flx/flx*^ mice, as previously described [35]. Consequently, HDL particles would be trapped within the fibrin meshwork together with other blood components. Finally, deletion of *Icmt* decreased ApoA1 protein abundance, which correlated with reduced vascular congestion, likely due to improved blood vessel perfusion, decreased body weight loss, and increased overall survival.

## Conclusions

This study reveals a complex role for ICMT in a mouse model of liver cancer. While *Icmt* deletion did not prevent tumor formation, it improved systemic health, reduced fibrosis and thrombosis, and prolonged survival. These results cast doubt on the utility of ICMT inhibition as a therapeutic strategy in HCC, although its potential in modulating the liver microenvironment and systemic complications remains promising.

## Supplementary Information

Supplementary Material 1.

Supplementary Material 2.

Supplementary Material 3.

Supplementary Material 4.

Supplementary Material 5.

## Data Availability

Transcriptomics data are available at the GEO under the accession number GSE297390. Proteomics data are available at the MassIVE database under accession number PXD064401 and PXD073274.
